# Impact of mydriasis in fluorescence lifetime imaging ophthalmoscopy

**DOI:** 10.1371/journal.pone.0209194

**Published:** 2018-12-28

**Authors:** SriniVas R. Sadda, Enrico Borrelli, Wenying Fan, Adel Ebraheem, Kenneth M. Marion, Soonil Kwon

**Affiliations:** 1 Department of Ophthalmology, David Geffen School of Medicine at UCLA, Doheny Eye Center, Los Angeles, CA; 2 Department of Ophthalmology, David Geffen School of Medicine at UCLA, Stein Eye Institute, Los Angeles, CA; 3 Department of Ophthalmology, Hallym Sacred Heart Hospital, Hallym University College of Medicine, Anyang, Gyeonggi, South Korea; Faculty of Medicine, Cairo University, EGYPT

## Abstract

Fluorescence lifetime imaging ophthalmoscopy (FLIO) is a novel technique that measures in vivo autofluorescence intensity decay over time of endogenous fluorophores in the retina. The Heidelberg Engineering FLIO system was used to obtain two 30 degree scans centered on the fovea of both eyes. The FLIO system uses a 473nm blue scanning laser light source and the emitted fluorescence is detected in two wavelengths channels, short and long spectral channels (SSC, LSC). Since the mydriatic status influence the FLIO result, the impact of mydriasis on FLIO need to be clarified. In this prospective, observational study, the impact of mydriasis on measurements from fluorescence lifetime imaging ophthalmoscope (FLIO) images in normal subjects were evaluated. 12 healthy participants (24 eyes) were volunteered and all subjects were scanned twice and the mean fluorescence lifetime (τ_m_) values were computed with dilation and without dilation on different days. Intraclass correlation coefficients (ICC) and coefficients of variation (CV) were calculated from the measured τ_m_ in dilated, nondilated and between the dilated and non-dilated setting. Test duration was also compared and correlated with lifetimes in both settings. Repeatability was excellent for both the dilation and non-dilation settings (ICC; 0.967–0.996; 0.926–0.986, respectively). The agreement between the dilation and non-dilation settings, however, were lower (ICC; 0.688–0.970). The τ_m_ in the non-dilation setting was significantly longer than in the dilation setting for the SSC (*P*<0.05). The FLIO test duration in the non-dilation setting was significantly longer than with dilation for the SSC (*P* <0.05). Although good repeatability in τ_m_ measurements between imaging sessions were observed both with and without dilation, the agreement was not as good when comparing dilated with non-dilated measurements. Since FLIO without mydriasis results in longer τ_m_ in the SSC and takes a longer time for image acquisition, maximal dilation is recommended for FLIO testing.

## Introduction

Fluorescence lifetime imaging ophthalmoscopy (FLIO) is a novel technique that measures in vivo autofluorescence intensity decay over time of endogenous fluorophores in the retina, such as lipofuscin, advanced glycation end products (AGE), collagen, and elastin [1–5]. Autofluorescence of the retina is excited by photons derived from laser light and the fluorescence lifetime is the average time between excitation and return of the tissue to its basal energy level by release of the long-wavelength photons [1]. Because fluorescence lifetime is specific for each fluorophore, the decay of fluorescence intensity after pulse excitation permits the differentiation of fluorophores with overlapping emission spectra. It can produce quantitative data based on the lifetimes of the different endogenous retinal fluorophores [1,6]. Standard FLIO image acquisition is usually performed with maximally dilated pupils to reduce the influence of the crystalline lens and to increase the time-correlated photon counts from retinal fluorophores [7]. Dysli et al. reported that the FLIO allows reproducible measurements of fluorescence lifetimes even with nondilated pupils. They also found that the agreement of mean fluorescence lifetimes between dilated and nondilated pupils were not as good when compared with the agreement between two measurements with nondilated pupils [1].

The repeatability of FLIO results under mydriatic conditions, however, has not been fully established, and is the subject of this report.

## Methods

### Subjects

In this prospective, observational, single-center study at the Doheny UCLA Eye Centers, we recruited 14 healthy, phakic volunteers aged 20 to 70 years. Subjects had no history of medical or ophthalmic disease, as confirmed by chart review and ophthalmic exam. Only subjects with clear crystalline lens were included after slit-lamp examination in this study. Written informed consent was obtained from all individuals prior to study participation. The study was approved by the Institutional Review Board of the University of California, Los Angeles and conducted in accordance with the ethical standards stated in the Declaration of Helsinki and in compliance with the regulations set forth by the Health Insurance Portability and Accountability Act.

### Study design

All subjects underwent detailed clinical examinations, including best corrected visual acuity (BCVA), intraocular pressure measurement, slit-lamp biomicroscopy, autorefraction using ARK-530 (Nidek Co. Ltd., Gamagori, Japan), ultrawidefield fundus photo using an Optos 200Tx (Optos, Dunfermline, UK), and optical coherence tomography using a Cirrus HD-OCT model 5000 with Angioplex^TM^ (Carl Zeiss Meditec, Dublin, CA, USA).

**Fig 1:**
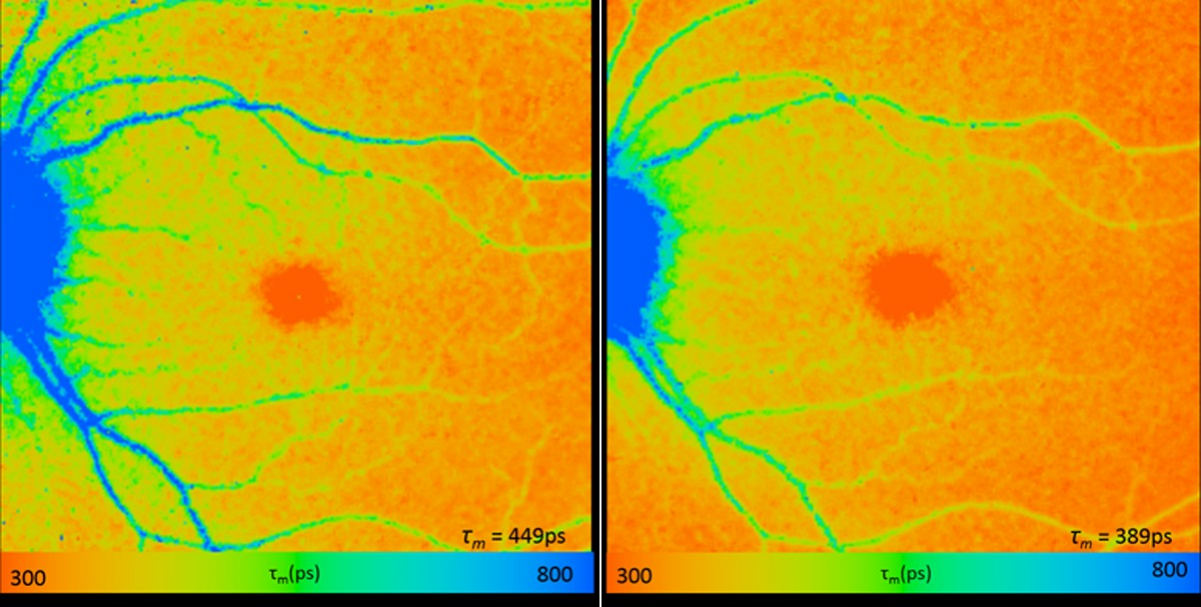
Fluorescence lifetime image of short spectral channel under non-dilated (left) and dilated (right) settings in same subject. Note that the longer lifetime pixels (green to blue in color) found in the more peripheral regions in the non- dilation setting compared to the dilated setting, and the overall longer τ_m_ (τ_m_ = 449 ps in without dilation, τ_m_ = 389 ps with dilation).

All subjects underwent FLIO measurement in both eyes with and without dilation using a prototype Heidelberg FLIO device (Heidelberg, Germany). FLIO measurements for evaluating repeatability were taken from each volunteer twice a day in a dark room with an interval of at least 1 hour between tests. Measurements always started with the right eye and then the left eye after a 5- minute break between eyes. FLIO testing with dilation and without dilation were performed on different day. Thus, in total, all subjects underwent four FLIO acquisitions in each eye. The pupil was dilated to at least 7mm in diameter using tropicamide 0.5% and phenylephrine HCl 2.5%. The pupillary diameter was measured by placing the pupil ruler as close as possible alongside the eye. All FLIO measurements were acquired by one operator (S.K.) (Fig 1).

### Setting for fluorescence lifetime imaging ophthalmoscopy

Basic principles of the FLIO technique have been describe in several reports [1,6,8]. In brief, the prototype FLIO was used for acquisition of fluorescence lifetime measurement in the retina with a 473 nm pulsed blue laser light at an 80 MHz repetition rate for excitation of retinal fluorescence. The fluorescence light was detected by highly sensitive hybrid photon-counting detectors in two different spectral channels (short spectral channel or SSC: wavelength 498–560 nm, long spectral channel or LSC: wavelength 560–720 nm) and registered by time-correlated single photon counting (TCSPC). At the same time, a confocal high-contrast infrared image was recorded by the system to record each photon at the correct spatial location. At least 1000 photons were collected per pixel for an adequate signal-to noise ratio.

### Data analysis

The software SPCImage 4.4.2 (Becker&Hickl GmbH, Berlin, Germany) was used for the analysis of fluorescence lifetime data. A three-exponential decay model was applied and used 3 x 3 pixel binning in both channels. The fluorescence decay can be allocated to three components, and thereby described through the lifetime parameters *τ*_*1~3*_ and amplitudes *a*_*1~3*_. For a global characterization of the fluorescence, an amplitude-weighted mean fluorescence lifetime value *τ*_*m*_ for each pixel was calculated as follows:

$$\tau_{m}=\left(\alpha 1*\tau 1+\alpha 2*\tau 2+\alpha 3*\tau 3\right)/\left(\alpha 1+\alpha 2+\alpha 3\right)$$

**Fig 2:**
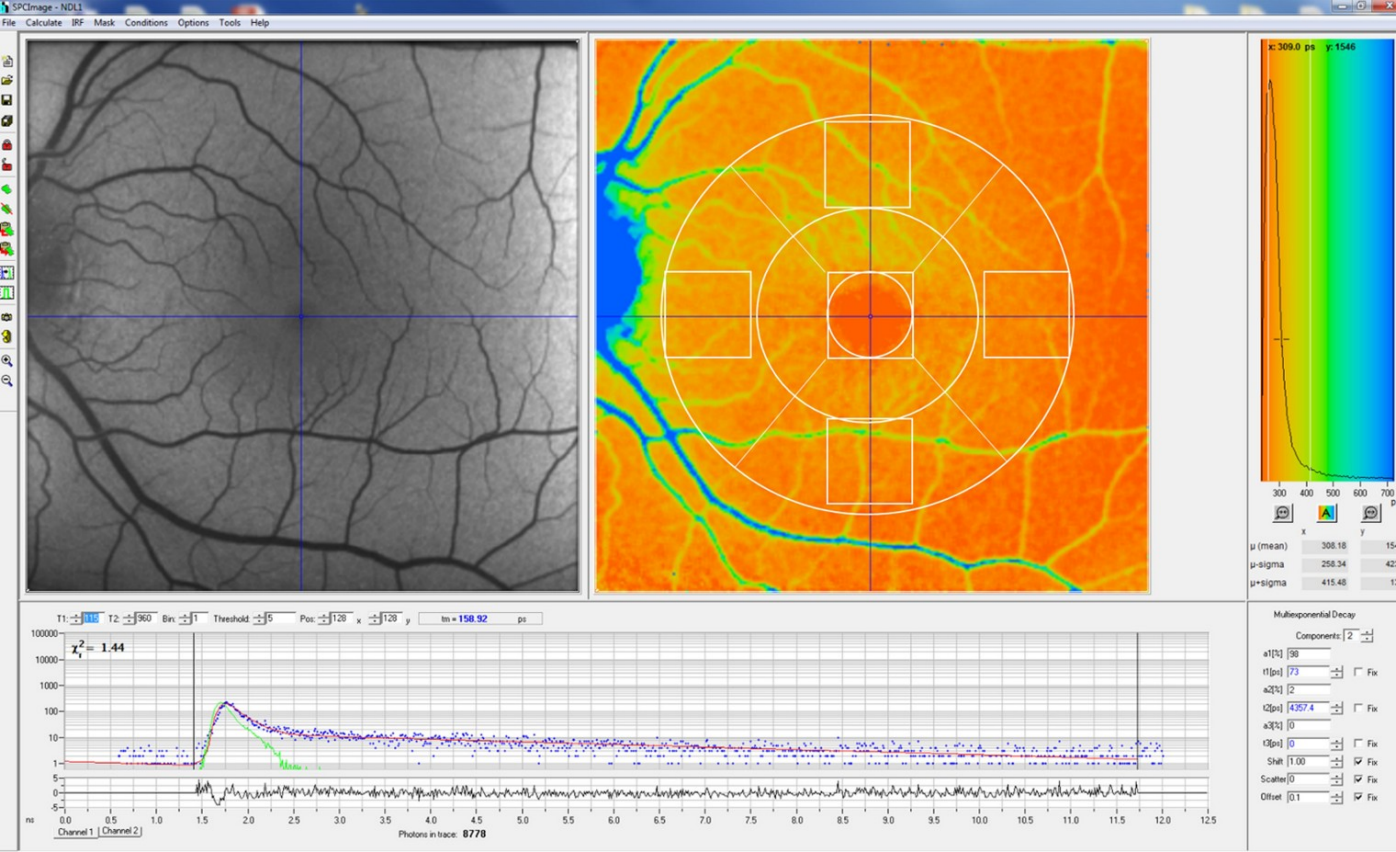
Example of a standard blue-light autofluorescence image (left) and fluorescence lifetime image of a normal subject with an overlaid ETDRS grid (right).

The “goodness” of the exponential fit of the exponential decay function was assessed by the chi-square value. For the analysis of different fundus regions, lifetime values were computed from five regions of interest (ROI) in the macular area which were derived from sectors of the ETDRS grid (each region consisting of a square 28 x 28 pixels in size): fovea, superior, inferior, nasal and temporal macula (Fig 2).

### Statistical analysis

Statistical analysis of the data was performed using SPSS 23.0 software (IBM corporation, NY).

To assess test-retest reliability, the *τ*_*m*_ of the entire macula and the 5 pre-specified regions were compared between the 1^st^ and 2^nd^ tests using intraclass correlation coefficients (ICC) and agreement was evaluated using Bland-Altman plots. In addition, for comparisons between tests under dilated and non-dilated settings, the agreement between measurements in dilated and non-dilated settings were also assessed by ICC and Bland-Altman plots. The differences in fluorescence lifetimes for the 5 ROIs between the 2 settings were investigated using the Mann Whitney U-test, and the inter-individual variability of fluorescence lifetime values were assessed by the coefficient of variation (CV). Test duration time of the 1^st^ and 2^nd^ measurement of both eyes was recorded and compared between the two settings. In addition, the difference in test duration between right and left eye, and between the 1^st^ and 2^nd^ measurements were analyzed by the Wilcoxon signed rank tests in both settings. The correlations of fluorescence lifetime with test duration time were also evaluated by Spearman’s correlation. Statistical results were expressed as *P*-values and a *P*-value of less than 0.05 was considered statistically significant.

## Results

Among the 14 volunteers, FLIO results from 2 subjects were excluded because of poor quality of images obtained without dilation. A total of 12 subjects with 24 eyes were included in the analysis. The mean age was 34.9 ± 5.1 years (range, 25–42) and 50% of the subjects were female. The mean spherical equivalent was -1.7 D ± 2.5, and -1.6 D ± 2.5 in the right and left eyes, respectively (Table 1).

**Table 1:** Demographics of the subjects.

**Number of Subject (eye)**	12 (24)
**Age**	34.9 ± 5.1
**Sex (M: F)**	6: 6
**Lens status (eyes)**	
Phakic: Pseudophakic	24: 0
**Spherical equivalent** **Mean ± SD (Diopter)**	
Right eye	-1.7 ± 2.5
Left eye	-1.6 ± 2.5

### Repeatability of FLIO with dilated and non-dilated setting

**Fig 3:**
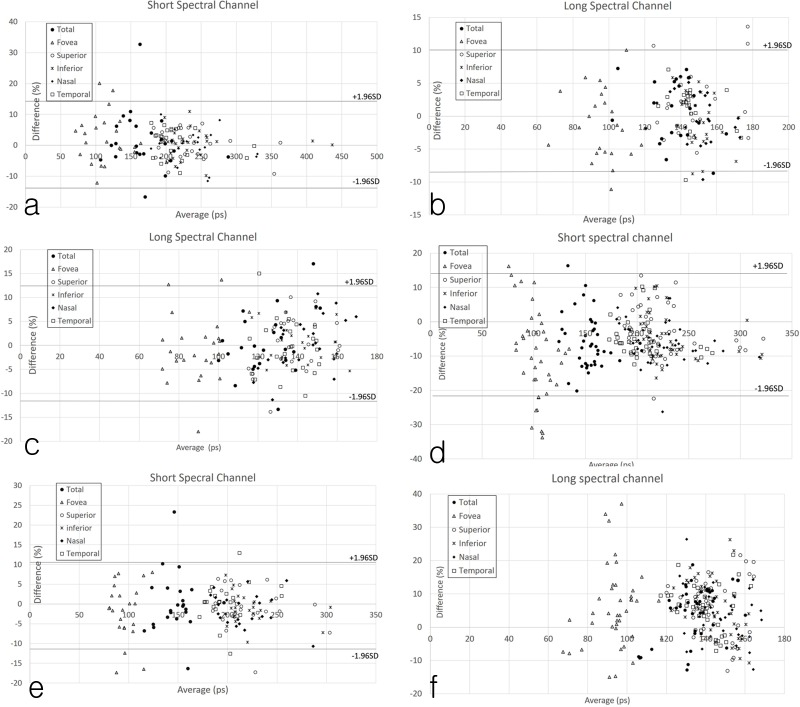
Bland-Altmans plot for repeatability of the 1^st^ and 2^nd^ measurements from the short spectral channel (SSC) and long spectral channel (LSC). SSC (a) and LSC (b) in the dilated setting, and SSC (c) and LSC (d) in the non-dilated setting. Bland-Altman plots comparing the measurements obtained in dilated and non-dilated setting for the SSC (e) and LSC (f) from both 1^st^ and 2^nd^ measurements. Individual points represent values for the total scan area as well as within specific macular regions.

**Fig 4:**
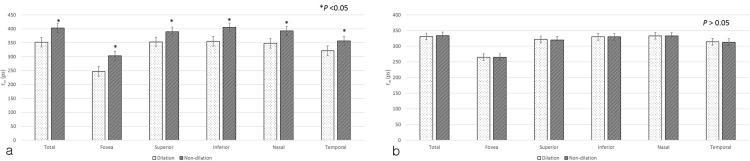
Comparison of mean fluorescence lifetimes from various pre-specified macular regions of both eyes dilated and non-dilated settings. (a; short spectral channel, b; long spectral channel, Mann-Whitney U-test, *P<0.05).

The repeatability of fluorescence lifetime between the 1^st^ and 2^nd^ measurements was evaluated from the entire scan area and from the 5 pre-specified ROI macular regions in dilated and non-dilated settings. The ICC of the *τ*_*m*_ between measurements for each setting is shown in Table 2. The repeatability of *τ*_*m*_ in both settings was excellent for both channels for both eyes (ICC; 0.967–0.996 in the dilated setting, ICC; 0.926–0.986 in the non-dilated setting). The agreement between the dilated and non-dilated setting was not as high as within each setting (ICC; 0.688–0.970). Fig 3 shows the combined Bland-Altman plots for the agreement of *τ*_*m*_ between two measurements in all ROI areas for both spectral channels in the dilated setting, non-dilated setting, and between the 2 settings. In the dilated setting, the *τ*_*m*_ measured for the entire scan area was 352.3ps ± 51.6 in the SSC and 331.1ps ± 23.8 in the LSC. The foveal area featured the shortest *τ*_*m*_ among the 5 ROIs in both spectral channels (246.7 ps ± 37.6 in SSC, 265.4 ps ± 17.5 in LSC). In the non-dilated setting, the *τ*_*m*_ was 403.4 ps ± 84.6 in the SSC, 334.4 ps ± 31.9 in the LSC, and 302.5 ps ± 66.6 in the SSC, 264.8 ps ± 26.8 in the LSC for entire scan area and foveal area, respectively. The *τ*_*m*_ from all other macular ROI regions in the non-dilated setting was statistically significantly longer than in the dilated setting for the SSC, while the LSC showed no significant differences between the 2 settings (Table 3 and Fig 4).

**Table 2:** The repeatability of mean fluorescence lifetime (τ_m_) of entire scan area and 5 pre-defined retinal areas in dilated and non-dilated settings.

	Channel	Rt. SSC	Rt. LSC	Lt. SSC	Lt. LSC
	Area	ICC (95% CI)	ICC (95% CI)	ICC (95% CI)	ICC (95% CI)
**Dilated**	**Total**	0.991 (0.966–0.998)	0.991 (0.966–0.998)	0.967 (0.887–0.991)	0.975 (0.914–0.993)
	**Fovea**	0.972 (0.895–0.992)	0.973 (0.898–0.993)	0.986 (0.952–0.996)	0.973 (0.906–0.992)
	**Superior**	0.989 (0.959–0.997)	0.996 (0.984–0.999)	0.981 (0.934–0.995)	0.990 (0.966–0.997)
	**Inferior**	0.988 (0.955–0.997)	0.992 (0.972–0.998)	0.994 (0.979–0.998)	0.981 (0.932–0.994)
	**Nasal**	0.983 (0.938–0.995)	0.979 (0.923–0.994)	0.977 (0.920–0.993)	0.976 (0.916–0.993)
	**Temporal**	0.992 (0.969–0.998)	0.981 (0.928–0.995)	0.994 (0.978–0.998)	0.990 (0.965–0.997)
**Non-dilated**	**Total**	0.981 (0.934–0.995)	0.972 (0.902–0.992)	0.956 (0.849–0.987)	0.953 (0.837–0.986)
	**Fovea**	0.950 (0.827–0.986)	0.966 (0.884–0.990)	0.926 (0.744–0.979)	0.952 (0.834–0.986)
	**Superior**	0.986 (0.951–0.996)	0.966 (0.883–0.990)	0.974 (0.910–0.993)	0.966 (0.881–0.990)
	**Inferior**	0.984 (0.943–0.995)	0.972 (0.902–0.992)	0.981 (0.935–0.995)	0.947 (0.817–0.985)
	**Nasal**	0.977 (0.921–0.993)	0.972 (0.901–0.992)	0.957 (0.851–0.988)	0.973 (0.905–0.992)
	**Temporal**	0.985 (0.948–0.996)	0.967 (0.884–0.990)	0.963 (0.873–0.989)	0.952 (0.833–0.986)
**Between Dilated and Non-dilated**	**Total**	0.883 (0.565–0.969)	0.911 (0.669–0.976)	0.876 (0.568–0.964)	0.953 (0.836–0.986)
	**Fovea**	0.801 (0.259–0.946)	0.736 (0.019–0.929)	0.821 (0.377–0.948)	0.875 (0.565–0.964)
	**Superior**	0.901 (0.633–0.973)	0.688 (0.213–0.877)	0.932 (0.762–0.980)	0.970 (0.896–0.991)
	**Inferior**	0.867 (0.505–0.964)	0.892 (0.599–0.971)	0.816 (0.362–0.947)	0.901 (0.658–0.972)
	**Nasal**	0.871 (0.519–0.965)	0.907 (0.655–0.975)	0.866 (0.535–0.961)	0.938 (0.785–0.982)
	**Temporal**	0.874 (0.532–0.966)	0.892 (0.600–0.971)	0.859 (0.511–0.959)	0.926 (0.743–0.979)

ICC, intraclass correlation coefficient; CI, confidence interval; Total: entire scan area

SSC, short spectral channel; LSC, long spectral channel

**Table 3:** The average and CV of mean fluorescence lifetime (τ_m_) of entire scan area and 5 pre-defined retinal areas in dilated and non-dilated setting.

	Area	Right eye		Left eye	
		SSC	LSC	SSC	LSC
		Mean ± SD (ps) CV	Mean ± SD (ps) CV	Mean ± SD (ps) CV	Mean ± SD (ps) CV
**Dilated**	**Total**	352.5 ± 46.9 13%	333.0 ± 23.5 7%	352.2 ± 56.7 16%	329.5 ± 24.4 7%
	**Fovea**	245.9 ± 33.8 14%	267.1 ± 17.0 6%	247.5 ± 41.5 17%	263.8 ± 18.2 7%
	**Superior**	350.6 ± 43.7 12%	322.8 ± 21.0 7%	355.3 ± 65.3 18%	321.3 ± 23.9 7%
	**Inferior**	354.4 ± 55.6 16%	331.9 ± 22.9 7%	355.1 ± 52.2 15%	327.8 ± 21.8 7%
	**Nasal**	342.3 ± 38.4 11%	333.2 ± 22.9 7%	352.6 ± 50.4 14%	332.0 ± 24.2 7%
	**Temporal**	321.2 ± 41.8 13%	315.9 ± 19.3 6%	320.4 ± 43.0 13%	312.0 ± 18.2 6%
**Non-dilated**	**Total**	392.5 ± 82.5 21%	332.7 ± 34.4 10%	414.3 ± 87.0 21%	336.0 ± 29.9 9%
	**Fovea**	292.0 ± 61.7 21%	263.4 ± 27.2 10%	313.0 ± 70.9 23%	266.2 ± 26.9 10%
	**Superior**	376.6 ± 68.5 18%	318.1 ± 29.3 9%	402.5 ± 88.1 22%	322.7 ± 29.5 9%
	**Inferior**	394.9 ± 111.9 28%	328.8 ± 35.6 11%	416.1 ± 111.7 27%	332.1 ± 32.2 10%
	**Nasal**	377.5 ± 71.8 19%	331.0 ± 32.2 10%	409.4 ± 85.2 21%	335.4 ± 31.6 9%
	**Temporal**	350.2 ± 72.0 21%	312.0 ± 28.4 9%	363.7 ± 77.1 21%	313.0 ± 26.1 8%

SSC, short spectral channel; LSC, long spectral channel; Total: entire scan area

SD, standard deviation; CV, coefficient of variation; ps: picosecond

The CV between measurements in the dilated setting were 11~18% for the SSC and 6~7% for the LSC. The CV in the non-dilated setting was higher than the dilated setting, particularly for the SSC (18~28% for the SSC compared with 8~11% for the LSC). Although the CV of the SSC was higher than that of the LSC in both settings, CV for the SSC was much higher in the non-dilated setting (Table 3).

### Test duration in dilated and non-dilated settings

**Fig 5:**
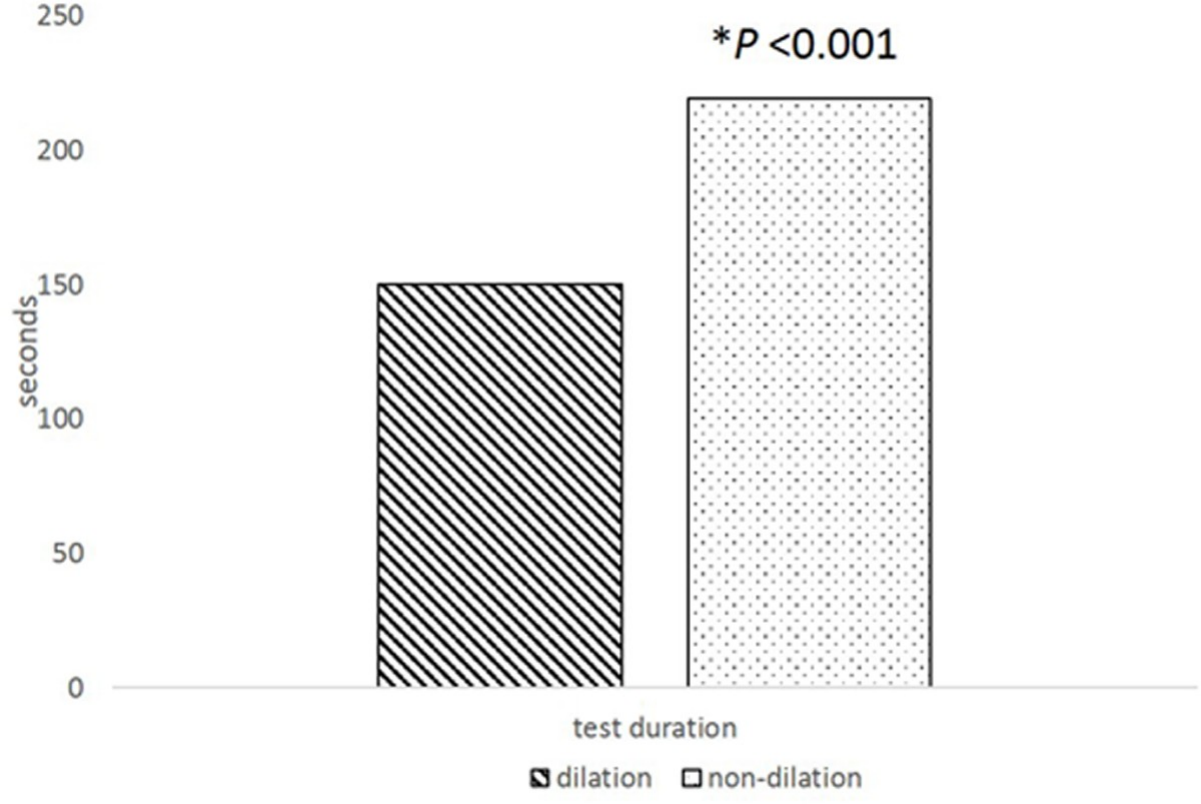
Difference in test duration time (seconds) in dilated and non-dilated settings. (Mann-Whitney U-test, *P<0.05).

The mean test duration time for FLIO acquisition with dilation was 146.4 sec ± 28.3 in the right eye and 148.3 sec ± 23.0 in the left eye for the 1^st^ measurement (Table 4). In contrast, without dilation the testing times (207.8 sec ± 53.6, 249.0 sec ± 61.4 for the right and left eyes, respectively) were significantly longer than with dilation (Mann-Whitney test, *P*<0.001, Fig 5).

**Table 4:** Test duration time of both eyes in two different settings.

	Right eye		Left eye	
	1^st^ measurement	2^nd^ measurement	1^st^ measurement	2^nd^ measurement
**Dilated setting**	146.4 sec ± 28.3	145.5 sec ± 24.4	148.3 sec ± 23.0	158.5 sec ± 23.9
**Non-dilated setting**	207.8 sec ± 53.6	193.8 sec ± 32.5	249.0 sec ± 61.4	225.0 sec ± 51.5

**Fig 6:**
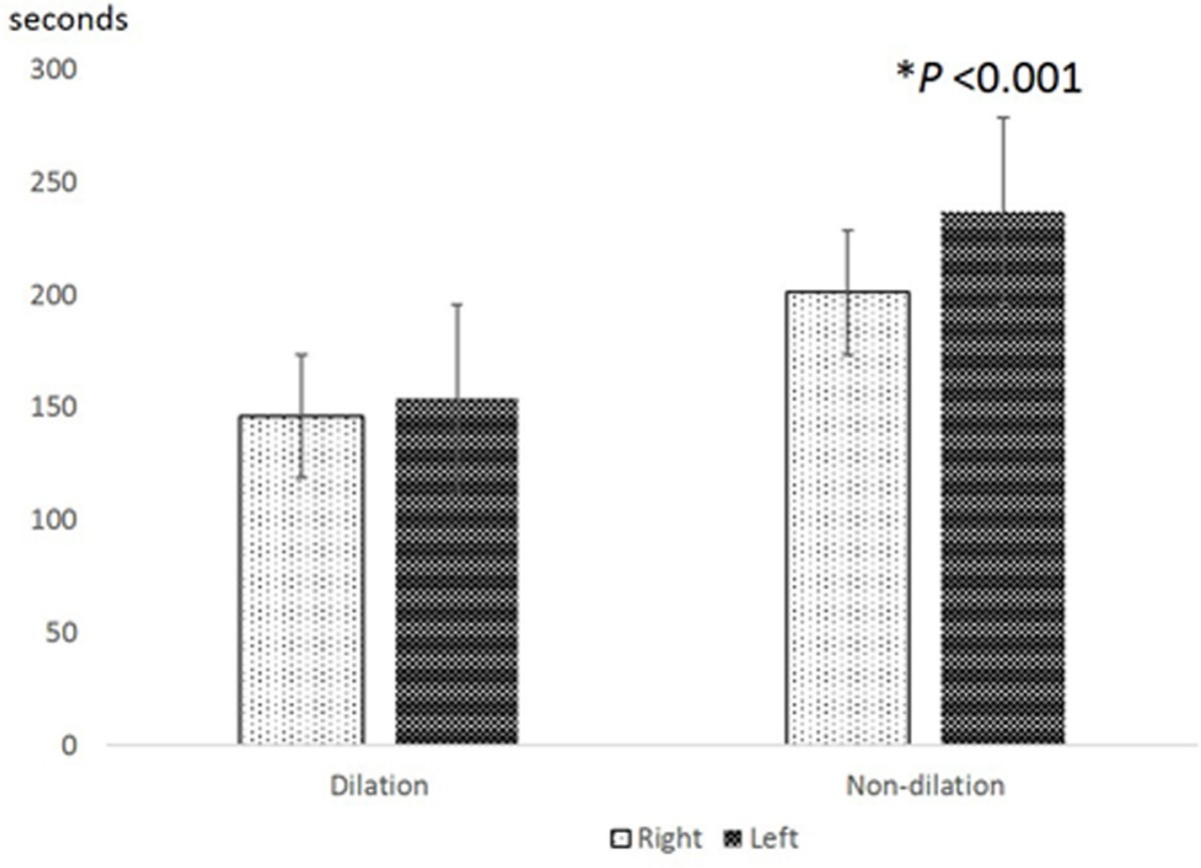
Difference in test duration time (seconds) between right and left eyes. (Wilcoxon signed rank test, *P<0.05).

**Fig 7:**
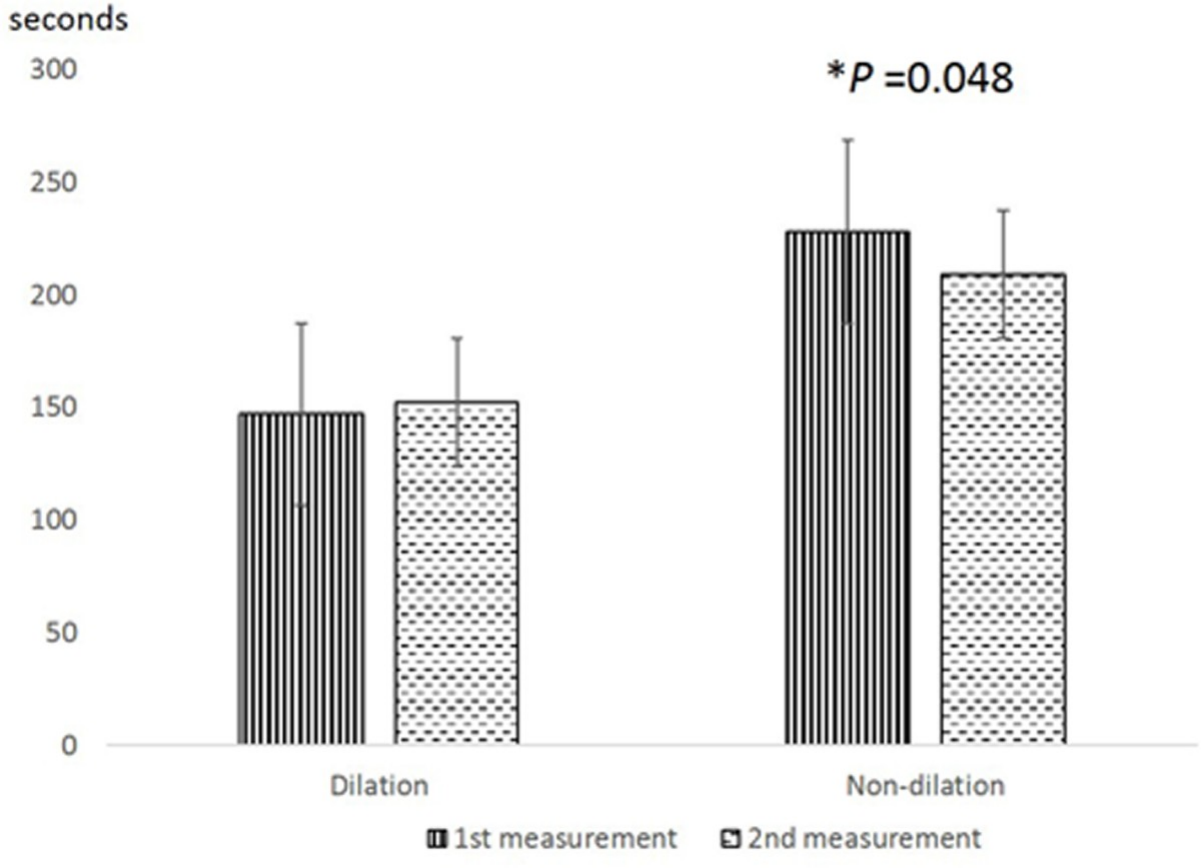
Difference in test duration time between the 1^st^ and 2^nd^ measurements of the fluorescence lifetime. (Wilcoxon signed rank test, *P<0.05).

There were no significant differences in test duration between the right and left eyes and between the 1^st^ and 2^nd^ measurements in the dilated setting, but it took significantly longer for the left eye (the second eye in the test sequence) than the right eye (Wilcoxon signed rank test, p<0.001, Fig 6). In the non-dilated setting, it took longer for the 1^st^ measurement than the 2^nd^ measurement later in the day (Wilcoxon signed rank test, p<0.001, Fig 7). However, there was no significant correlation between test duration and fluorescence lifetimes in either the dilated or non-dilated settings (Spearman’s correlation, P>0.05).

## Discussion

FLIO is a novel imaging technique with many suggested retinal disease applications including in early stage diabetic retinopathy, Alzheimer’s disease, Stargardt disease, and age-related macular degeneration [6,9–11]. Because each retinal fluorophore possesses a characteristic fluorescence lifetime, which is also influenced by its environment, fluorescence lifetime measurements provide additional information compared with conventional fluorescence imaging based on intensity alone. Thus, the detection of subtle changes and discrimination of various retinal fluorophores might be helpful in detecting early functional alterations and can be employed as diagnostic tools in retinal diseases. Dysli et al. evaluated the reproducibility of FLIO fluorescence lifetimes in the macula of healthy subjects [1]. They evaluated the repeatability of two FLIO measurements with nondilated pupils and correlated these measurements with a 3^rd^ FLIO measurement obtained through a dilated pupil. They reported that the correlation of mean fluorescence lifetime values between dilated and non-dilated settings was not as good as the correlation between the two undilated FLIO measurements. Our results under non-mydriatic conditions were overall consistent with those of Dysli and colleagues [1]. Specifically, FLIO repeatability with non-dilated pupils was good but the values were different compared with mydriasis. The repeatability of FLIO in the dilated setting itself, however, was not assessed in Dysli’s report, nor was the repeatability in the dilated setting compared to the non-dilated. This is an important aspect to characterize as most FLIO measurements from various studies have thus far been obtained with full dilation [1,6,9–11], though the optimal conditions under which FLIO testing should be performed have not been established. In our study, the repeatability of FLIO measurements with mydriasis was excellent and was better than that obtained with undilated pupils (Table 2). We also observed that FLIO acquisition times were longer with undilated pupils. Using the FLIO technique, autofluorescence of the retina is excited with a laser impulse and the excited fluorophore emits a long-wavelength photon while returning to its ground level. Since the laser and the photons comes in and out through the pupil, it is perhaps not surprising that it takes a longer time without dilation to collect enough photons through a smaller pupil aperture (Fig 5 and Table 4). Longer acquisition time and inevitable eye movements during measurements may cause more erroneous pixels, which the instrument displays as blue dots with a higher chi-square value, in the peripheral area, even though eye tracking system adjusted for the spatial location. These factors could delay the fluorescence lifetime and yield a larger CV in the non-mydriatic setting than with mydriasis.

Among the 14 volunteers, 2 volunteers were excluded due to poor quality of images which were blurry and featured a very prolonged long testing time–this only occurred in the non-dilated setting. We attempted to re-scan these two individuals on a second day, but failed again. We assumed that the excessive pupil reaction of non-dilated pupil caused this blurry images and long test duration. All of these findings support the support the use of mydriasis for FLIO testing in future studies.

Interestingly, despite these issues, significant differences in *τ*_*m*_ between the dilated and non-dilated settings were only found in the SSC, and not in the LSC (Fig 4). The findings of Schweitzer et al. may have explained this phenomenon, as the observed that the influence of the lens was larger on fluorescence lifetimes when the pupil was constricted [2]. The fluorescence of crystalline lens has been shown to have a relatively long lifetime especially in the short-wavelength channel. Thus, a significant effect of mydriasis on *τ*_*m*_ only in the SSC can be explained by the larger impact of the crystalline lens on the SSC when the pupil is not dilated.

Pupil aperture effects could also explain the right–left eye differences observed in our study in the non-dilated setting. As our acquisition protocol always started with the right eye first, consensual pupillary adaptation following prolonged bright light exposure to the fellow eye may have yielded a more constricted pupil when testing the undilated left eye, resulting in a longer scanning time (Fig 6). Moreover, longer scanning times are more likely to result in more erroneous pixels (due to small tracking failures), which could potentially lead lower repeatability and elongated fluorescence lifetimes in the non-dilated setting. Although we did observe a somewhat lower repeatability in the left eye in the undilated setting (Table 2), we did not observe and correlation between test duration and fluorescence lifetime. Of note, the acquisition time was shorter for the 2^nd^ measurement compared to the 1^st^ measurement in the non-dilated setting. We suspect patient cooperation may have been a factor. As the acquisition light is quite bright, subjects may have been more tolerant during the second testing session when they knew what brightness or discomfort to expect. Better cooperation and more stable fixation would reduce the time needed to collect the required number of photons.

Our study has some limitations which should be considered when assessing our results. First our sample size is relatively small, and thus underpowered to detect small differences in measurements between acquisition attempts. Second, although we confirmed that all pupils following dilation achieved a diameter of at least 7 mm, we could not adjust for differences in non-mydriatic pupil size between individuals.

In summary, although the repeatability of FLIO was good with and without dilation, it was better with dilation. Importantly, FLIO measurements with and without dilation were not as well correlated between each other. Without dilation, FLIO acquisition took longer and measured fluorescence lifetimes were longer, particularly in the SSC, likely reflecting the impact of the crystalline lens. FLIO has proved its great potential for early diagnosis and detecting subtle changes in various retinal diseases through many studies. Based on our findings, maximal dilation should be strongly considered for all future FLIO studies.

## Supporting information

S1 Dataset Demographics of the subjects, the values of mean fluorescence lifetime (τm) of each area and the test duration in dilation and non-dilation setting. (XLSX)
